# The Knee-Jerks in Pneumonia

**Published:** 1904-10-15

**Authors:** 


### THE KNEE-JERKS IN PNEUMONIA.

The differential diagnosis in many of the febrile conditions met with in children is often a clinical problem which taxes even the most experienced and capable practitioner, and any help in this direction is sure of wide appreciation. Probably an examination of the state of the knee-jerks in such circumstances is not usually practised, yet there are numerous observations on record which show that this examination might with advantage be included in the usual scheme of clinical investigation. M. Pfaundler[1] has drawn attention to the frequent absence or diminution of the knee-jerks in children suffering from croupous pneumonia. He observed this in 27·5 per cent. of the cases coming under observation. The loss was noted as a rule when the temperature was high, but at times it was seen after the fever had subsided. The cases in which the knee-jerks were absent were for the most part severe ones, and were marked by the early appearance of cerebral symptoms. Further, in some instances the absence of the jerks was observed prior to the development of physical signs of pneumonia. It was never seen in cases of lobular pneumonia. This observation is of special interest in connection with an experience put on record some years ago by Dr. Hughlings Jackson,[2] who described absence of the knee-jerks in three cases of croupous pneumonia. In one patient—a man of 23 years—there were certain peculiarities in the actions of the respiratory muscles, from which it was concluded that there was some local morbid change in the thoracic spinal cord. The muscles did not act in respiration proper, but they did so in the voluntary movement of so-called "forced inspiration." The patient made a good recovery, and the knee-jerks returned. In reference to the absence, as above described, of the knee jerks in the croupous pneumonia of children, it is suggested that this fact may be of value in distinguishing that disease from meningitis. But this is certainly a distinction that cannot be relied on as an absolute rule. For not only are there cases of pneumonia in which the knee-jerks retain their normal activity, but, on the other hand, spinal meningitis and even cerebral meningitis may lead to suppression of the jerks. This latter fact was also described by Dr. Hughlings Jackson. Dr. Buzzard[3] considers that such absence may be due to an extension of the inflammation to the spinal meninges, and thus to an affection of the roots of the spinal nerves, but however explained, it is certain that in some cases which in their clinical aspects are purely cases of cerebral meningitis the knee-jerks cannot be obtained. Dr. Lees[4] describes this condition in cases of tubercular meningitis and says that excepting some cerebellar tumours this is the only intra-cranial lesion which abolishes the knee-jerk, though its absence for a time may be noted after an apoplectic or an epileptic seizure. The suggestion has been made that such absence may be an aid in the attempt to distinguish between tubercular meningitis and enteric fever. A sustained pyrexia in a child frequently demands the discussion of these two possibilities. But here again it would appear that an absolute reliance on the suggestion just referred to cannot be advised. Thus according to Remlinger,[5] in 100 cases of typhoid fever the knee-jerks were absent in 29 instances. The same authority concludes that such absence on the whole is a sign favourable rather than otherwise. On the other hand, in severe adynamic forms of the disease the tendon phenomena tend to be exaggerated and ankle clonus and patellar clonus may be observed. In convalescence there is always more or less exaggeration of the knee-jerks. Another febrile condition in which the knee-jerks may possibly be diminished or lost is diphtheria. That this is the case when evidences of post-diphtheritic paralysis are present is well known; but, according to Dr. Walker Downie,[6] the loss may be an early event, and may be an aid in the diagnosis shortly after the onset of the disease. Thus there is clinical evidence that the knee-jerks may be absent in a number of widely differing pyrexial diseases in childhood, and therefore it cannot be claimed that the observation taken alone has a very exact significance. But in any given case the clinical associations may render the diagnostic issue a very narrow one, and an observation on the state of the knee-jerks may be of considerable value in turning the scale in one direction or the other.

[1] Brit. Med. Jour. (Epitome), January 17, 1903. [2] Lancet, December 22, 1894. [3] Clinical Jour., vol. ii., p. 145. [4] Ibid., p. 162. [5] Brit. Med. Jour. (Epitome), March 23, 1901. [6] Transactions of the Glasgow Medico-Chirurgical Society, vol. i.

